# Supplementary material for: MetaQTL: a package of new computational methods for the meta-analysis of QTL mapping experiments
Source: BMC Bioinformatics. 2007 Feb 8;8:49. doi: 10.1186/1471-2105-8-49 (PMC1808479; doi:10.1186/1471-2105-8-49)
Supplement: Additional File 7 — MetaQTL Package : jar file and tutorial. This Zip archive contains both the MetaQTL JAR file and the files of the tutorial. [file 1471-2105-8-49-S7.zip › org.inra.metaqtl/doc/org/metaqtl/main/class-use/CmdLineParser.UnknownOptionException.html]

Uses of Class org.metaqtl.main.CmdLineParser.UnknownOptionException


|  |  |  |
| --- | --- | --- |
| **Overview** \| **Package** \| **Class** \| **Use** \| **Tree** \| **Deprecated** \| **Index** \| **Help** | |  |
| PREV   NEXT | **FRAMES**    **NO FRAMES**     **All Classes** |  |


---


## **Uses of Class org.metaqtl.main.CmdLineParser.UnknownOptionException**

| Packages that use CmdLineParser.UnknownOptionException | |
| --- | --- |
| **org.metaqtl.main** |  |

Uses of CmdLineParser.UnknownOptionException in org.metaqtl.main

| Subclasses of CmdLineParser.UnknownOptionException in org.metaqtl.main | |
| --- | --- |
| `static class` | `CmdLineParser.NotFlagException`             Thrown when the parsed commandline contains multiple concatenated short options, such as -abcd, where one or more requires a value. |
| `static class` | `CmdLineParser.UnknownSuboptionException`             Thrown when the parsed commandline contains multiple concatenated short options, such as -abcd, where one is unknown. |

| Methods in org.metaqtl.main that throw CmdLineParser.UnknownOptionException | |
| --- | --- |
| `void` | `CmdLineParser.parse(java.lang.String[] argv)`             Extract the options and non-option arguments from the given list of command-line arguments. |
| `void` | `CmdLineParser.parse(java.lang.String[] argv, java.util.Locale locale)`             Extract the options and non-option arguments from the given list of command-line arguments. |

---


|  |  |  |
| --- | --- | --- |
| **Overview** \| **Package** \| **Class** \| **Use** \| **Tree** \| **Deprecated** \| **Index** \| **Help** | |  |
| PREV   NEXT | **FRAMES**    **NO FRAMES**     **All Classes** |  |


---
